# The Association of Dietary Patterns with High-Risk Human Papillomavirus Infection and Cervical Cancer: A Cross-Sectional Study in Italy

**DOI:** 10.3390/nu10040469

**Published:** 2018-04-11

**Authors:** Martina Barchitta, Andrea Maugeri, Annalisa Quattrocchi, Ottavia Agrifoglio, Aurora Scalisi, Antonella Agodi

**Affiliations:** 1 Department of Medical and Surgical Sciences and Advanced Technologies “GF Ingrassia”, University of Catania, via S. Sofia, 87, 95123 Catania, Italy; martina.barchitta@unict.it (M.B.); andreamaugeri88@gmail.com (A.M.); annalisaquattrocchi@hotmail.com (A.Q.); ottavia.agrifoglio@gmail.com (O.A.); 2 Unità Operativa di Screening Ginecologico, Azienda Sanitaria Provinciale di Catania, 95126 Catania, Italy; aurora.scalisi@aspct.it

**Keywords:** cervical intraepithelial neoplasia, Mediterranean diet score, principal component analysis, Western diet, prudent diet

## Abstract

Specific foods and nutrients help prevent the progression of persistent high-risk human papillomavirus (hrHPV) infection to cervical cancer (CC). The aim of this study was to investigate dietary patterns which may be associated with hrHPV status and the risk of high-grade cervical intraepithelial neoplasia (CIN2+). Overall, 539 eligible women, including 127 with CIN2+, were enrolled in a cross-sectional study, and tested for hrHPV infection. Food intake was estimated using a food frequency questionnaire. Logistic regression models were applied. Using the Mediterranean Diet Score, we demonstrated that, among 252 women with a normal cervical epithelium, medium adherence to the Mediterranean diet decreased the odds of hrHPV infection when compared to low adherence (adjOR = 0.40, 95%CI = 0.22–0.73). Using the principal component analysis, we also identified two dietary patterns which explained 14.31% of the variance in food groups intake. Women in the third and fourth quartiles of the “Western pattern” had higher odds of hrHPV infection when compared with first quartile (adjOR = 1.77, 95% CI = 1.04–3.54 and adjOR = 1.97, 95%CI = 1.14–4.18, respectively). Adjusting for hrHPV status and age, women in the third quartile of the “prudent pattern” had lower odds of CIN2+ when compared with those in the first quartile (OR = 0.50, 95%CI = 0.26–0.98). Our study is the first to demonstrate the association of dietary patterns with hrHPV infection and CC and discourages unhealthy habits in favour of a Mediterranean-like diet.

## 1. Introduction

Cervical cancer (CC) is one of the most common types of cancer in females worldwide, second only to breast cancer [1]. Prediction models show that without innovative preventive strategies, the number of cases of CC will rise to 702,500, an alarming increase of 42% [2]. Vaccination and safe sex education have been established as effective prevention strategies against progression from persistent human papillomavirus (HPV) infection to cervical intraepithelial neoplasia (CIN) and/or carcinoma in situ [3]. However, the natural history of CC hints at the possibility of also achieving prevention through nutrient-mediated programs which lead to modifications of the host’s immune system [4]. These strategies should be developed to stimulate a host cell-mediated immune response against the HPV oncogenic proteins, E6 and E7 [3]. Epidemiological studies have revealed that several food groups or nutrients could prevent the progression of precursor lesions to CC. Results from the European Prospective Investigation into Cancer and Nutrition (EPIC) study showed a significant inverse association between fruit daily intake and CC [5]. In particular, fruit and vegetable consumption, as well as the intake of nutrients (i.e., vitamins A, C, and E, folates, carotenoids, and minerals), has been associated with a reduced risk of HPV infection, CIN and CC [6,7,8,9,10,11,12,13,14]. These findings support the role of vitamins as protective nutrients against CC, via the inhibition of cancer cell proliferation [15], the stabilization of p53 [16], the prevention of DNA damage and the reduction of immune suppression [15,17]. 

Although previous studies have generally focused on the consumption of specific food groups and/or nutrients, intervention programs based on dietary patterns, rather than on single nutrients or food groups, may be more effective in reducing the risk of HPV infection and CC. Since a lack of evidence exists regarding the synergic effects of food groups on CC etiopathology, we aimed to identify both *a priori* and *a posteriori* dietary patterns which may be associated with HPV status and high grade CIN risk.

## 2. Materials and Methods 

### 2.1. Study Design

The study protocol, approved by the ethics committee of the involved Institution (CE Catania 2; Prot. N. 227/BE and 275/BE) and performed according to the Declaration of Helsinki, has been fully described elsewhere [18]. Briefly, from 2013 to 2015, all women diagnosed with an abnormal Papanicolaou (PAP) test who had not had treatment and were referred to the cervical cancer screening unit (Unità Operativa di Screening Ginecologico) at the Azienda Sanitaria Provinciale (ASP), in Catania (Italy) for further examination by colposcopy and biopsy, were invited to participate in a cross-sectional study. Participants were fully informed of the purpose and procedures of the study, and a signed written consent was obtained. Women were classified by histological diagnosis and tested for high-risk HPV (hrHPV16, 18, 31, 33, 35, 39, 45, 51, 52, 56, 58, 59, and 68) using digene HC2 HPV DNA Test (Qiagen, Milan, Italy). Thus, women were classified as hrHPV positive if they were infected with any of the thirteen hrHPV types; otherwise, women were classified as hrHPV negative. Notably, the specific HPV genotype was not provided by the test. According to the histological results, women were further classified as cases (CIN2+: CIN2, CIN3 or carcinoma in situ-CIS) or controls (≤CIN1: CIN1 or normal cervical epithelium). A structured questionnaire was used by trained epidemiologists to obtain information on sociodemographic variables and lifestyle factors. Women were classified into two categories of educational level: low–medium (primary school, i.e., ≤8 years of school) and high education level (high school education or greater, i.e., >8 years of school). Body mass index (BMI) and nutritional status were calculated based on criteria from the World Health Organization [19].

### 2.2. Dietary Assessment

Dietary data were obtained by a 95-item semi-quantitative Food Frequency Questionnaire (FFQ), using the previous month as the reference period [20]. For each food item, women were asked to report the frequency of consumption and portion size. To estimate the amount of each food item and to minimize inaccuracies, an indicative photograph atlas was used. Frequencies of food consumption were classified into twelve categories, ranging from “almost never” to “two or more times a day”. The medium serving sizes were described by natural portions or standard weight and volume measures of the servings commonly consumed in the Italian population. Accordingly, portion size was classified into three categories: small (half a medium serving size), medium, and large (1.5 times or more than a medium serving size). The food intakes derived from the FFQ were calculated by multiplying the frequency of consumption with the daily portion size of each food group. The total energy intake was calculated using the United States Department of Agriculture (USDA) Nutrient Database (http://ndb.nal.usda.gov) adapted to Italian food consumption patterns. Food intakes were adjusted for total energy intake using the residual method [21]. 

### 2.3. The Mediterranean Diet Score

Adherence to Mediterranean diet (MD) was assessed using the Mediterranean Diet Score (MDS) [22,23]. This *a priori* score includes 9 components: fruits and nuts, vegetables, legumes, cereals, lipids, fish, dairy products, meat products, alcohol and the ratio of unsaturated to saturated lipids. For components that are more consumed in Mediterranean countries (vegetables, legumes, fruits and nuts, cereals, fish, and a high ratio of unsaturated to saturated lipids), women whose consumption was below or equal to the median value of the population were assigned a value of 0, and a value of 1 was assigned otherwise. For components consumed less frequently in Mediterranean countries (dairy and meat products), women whose consumption was below the median were assigned a value of 1, and a value of 0 was assigned otherwise. A value of 1 was given to women consuming a moderate amount of alcohol (5 to <25 g per day). The MDS ranges from 0 (no-adherence) to 9 (perfect adherence). MD adherence was categorized, according to the MDS, as follows: low adherence (MDS range: 0–3), medium adherence (MDS range: 4–6), or high adherence (MDS range: 7–9) [24]. Low adherence to MD was used as the reference for further analyses.

### 2.4. Principal Component Analysis

*A posteriori* dietary patterns were extracted using principal component analysis (PCA). We firstly classified the 95 FFQ food items into 39 predefined food groups, based on the similarity of nutrient profiles or culinary usage. Individual food items were preserved if they constituted a distinct item on their own (e.g., eggs, pizza, coffee or tea, etc.) or if they were thought to represent a particular dietary pattern (e.g., wine, alcoholic drinks, and chips, etc.). For each food group, the energy-adjusted variable was entered into the factor analysis. Factors were rotated by orthogonal transformation (varimax rotation) to maintain uncorrelated factors and to facilitate interpretability. The number of retained dietary patterns was determined according to eigenvalues (eigenvalues >2.0), scree plot examination, and interpretability. Factor loadings with an absolute value ≥0.2 were retained to define food groups that characterized dietary patterns. To confirm internal reproducibility, a factor analysis was performed separately in two randomly selected subgroups, using the same approach as for the main analysis. Factor scores for each dietary pattern were computed as the sum of products between observed energy-adjusted food group intakes and their factor loadings. For each dietary pattern, factor scores were categorized by quartiles (Q1–Q4); the lowest quartile (Q1) of each dietary pattern was used as the reference for further analyses.

### 2.5. Statistical Analyses

Statistical analyses were performed using SPSS software (version 22.0, SPSS, Chicago, IL, USA). Descriptive statistics were used to characterize the population using frequencies and means ± standard deviations (SDs). The two-tailed Chi-squared test was used for the statistical comparison of proportions, whereas continuous variables were tested using Student’s *t* tests. Trends across dietary pattern categories were analyzed using generalized linear models for continuous variables and Chi-squared tests for categorical variables. Unconditional multiple logistic regression models were used to estimate odds ratios (ORs) and corresponding 95% confidence intervals (95%CI) of hrHPV infection and CIN2+ status associated with PCA-derived dietary patterns, adherence to MD and to each MDS component, as well as with one-unit increase in factor scores and MDS. ORs and 95%CIs for hrHPV infection were calculated among women with a normal cervical epithelium using the following models: age-adjusted model (Model 1); model adjusted for variables found to be significantly associated with hrHPV infection in univariate analysis (Model 2). ORs and 95%CIs for CIN2+ status were calculated among women classified as cases or controls using the following models: age-adjusted model (Model 1); a model adjusted for age and hrHPV status (Model 2). All statistical tests were 2-sided, and *p*-values less than 0.05 were considered statistically significant.

## 3. Results

### 3.1. Study Population

Overall, 539 women with abnormal PAP test were classified by histological diagnosis and tested for hrHPV. Among these, 252 were diagnosed with a normal cervical epithelium (46.7%), 160 were diagnosed with CIN1 (29.7%), 57 with CIN2 (10.6%), 67 (12.4%) with CIN3 and 3 (0.6%) with CIS. With regard to hrHPV status, women were classified as hrHPV positive (hrHPV+; *n* = 302; 56%) or hrHPV negative (hrHPV–; *n* = 237; 44%). Table 1 displays the characteristics of women diagnosed with a normal cervical epithelium according to hrHPV status. In particular, the odds of being diagnosed with hrHPV infection increased among younger women (≤median age) (OR = 2.4; 95%CI = 1.0–5.8; *p* = 0.043), smokers (OR = 2.6; 95%CI = 1.1–6.2; *p* = 0.035), underweight–normal weight women (OR= 3.2; 95%CI= 1.1–9.5; *p* = 0.028) and nulliparous women (OR = 4.9; 95%CI = 1.7–14.1; *p* = 0.002). Table 2 shows the characteristics of women according to case/control classification. The odds of being diagnosed with CIN2+ were greater among younger women (≤median age) (OR = 2.3; 95%CI = 1.6–3.5; *p* = 0.001), smokers (OR = 2.0; 95%CI = 1.3–3.0; *p* = 0.001), and nulliparous women (OR = 1.7; 95%CI = 1.1–2.6; *p* = 0.011), as well as among oral contraceptive users (OR = 1.9; 95%CI = 1.0–3.5; *p* = 0.039). However, after adjustment for hrHPV status, no statistically significant differences between cases and controls were evident.

### 3.2. Dietary Assessment

**Figure 1:**
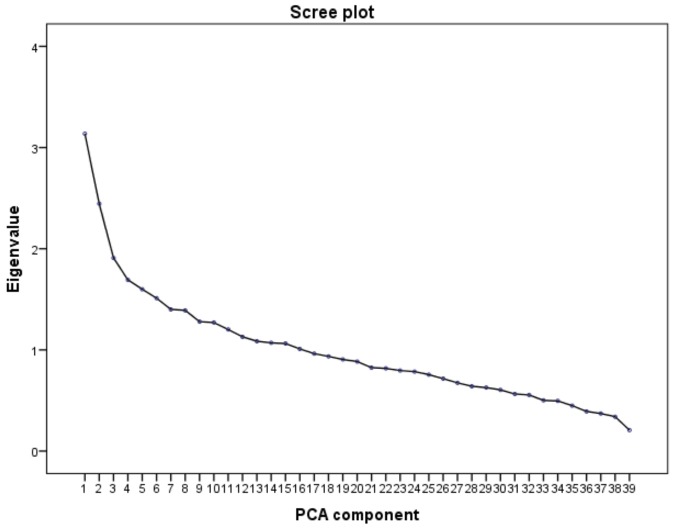
Scree plot of the eigenvalues. The scree plot, used to determinate the appropriate number of principal components, shows the eigenvalues, which represent the partitioning of the total variation accounted for by each principal component against the PCA component number.

**Figure 2:**
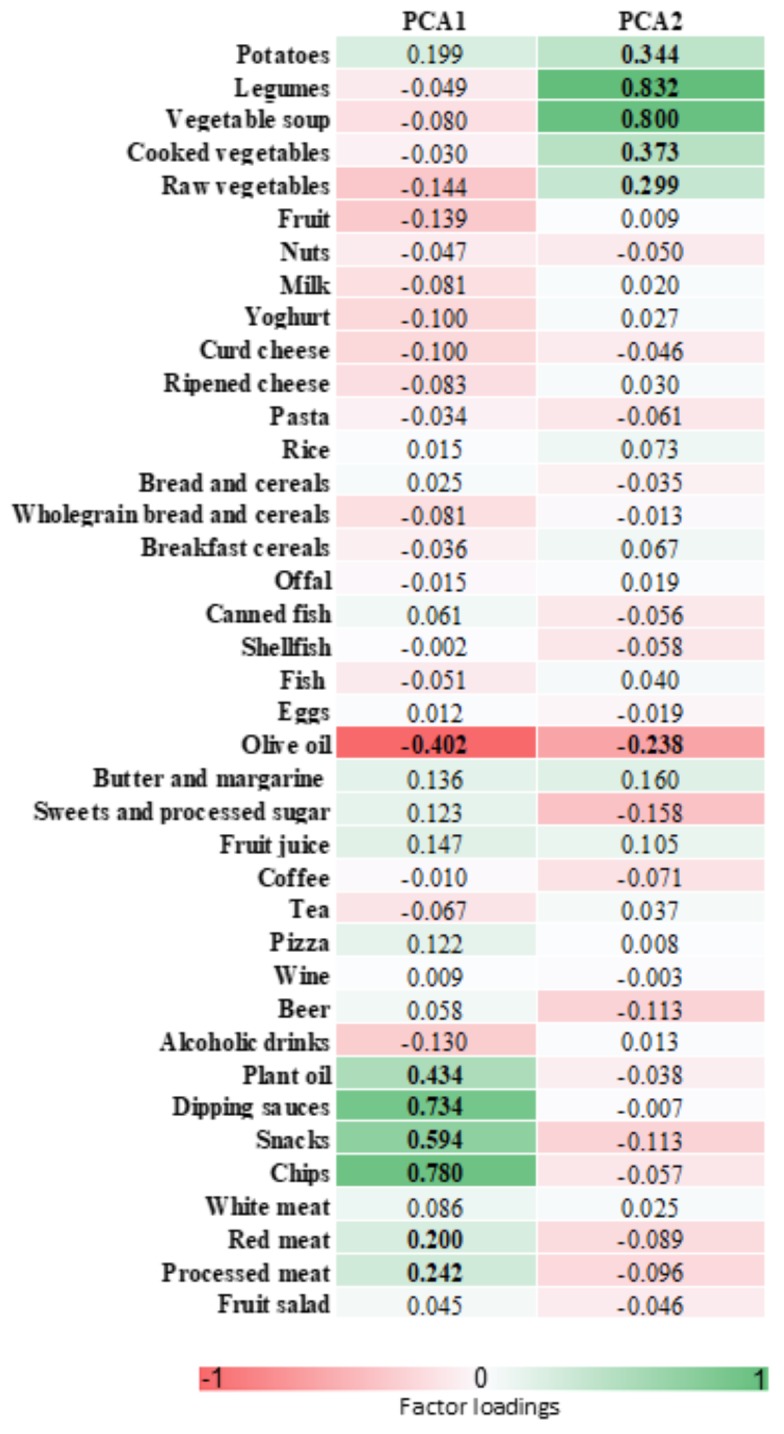
Table of factor loadings that characterize each dietary pattern. In red are food groups that negatively characterize dietary patterns; in green are food groups that positively characterize dietary patterns), factor loadings ≥0.2 are in bold font. PCA1: Western dietary pattern; PCA2: prudent dietary pattern.

**Figure 3:**
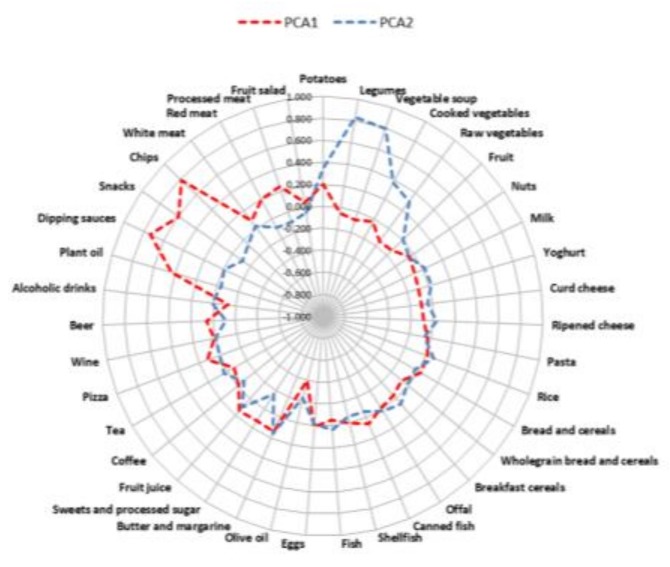
Radar graph of factor loadings that characterize each dietary pattern. The red line represents the distribution of the factor loadings related to the PCA1 component (Western dietary pattern). The blue line represents the distribution of factor loadings related to the PCA2 component (prudent dietary pattern).

The mean MDS value was 4.2 (median, 4; range, 0–9). According to the MDS, women were classified as follows: 33.1% had low adherence; 60.0% had medium adherence; and 6.9% had high adherence. No statistically significant differences in sociodemographic characteristics were observed across categories of the MD pattern. Based on scree plot examination (Figure 1), we identified two major dietary patterns with eigenvalues ≥2.0 which explained 14.31% of the total variance among 39 food groups. The first dietary pattern, named “Western”, was positively characterized by a high intake of chips, snacks, dipping sauces, plant oils, processed and red meats, with low intake of olive oil. The second one, named “prudent”, consistent with the well-accepted term used in this field of research, was positively characterized by a high intake of legumes, vegetable soups, potatoes, cooked, raw vegetables and olive oil (Figure 2 and Figure 3). The distribution of population characteristics by dietary pattern category is reported in Table 3. A high Western dietary pattern score was associated with younger age and smoking. No statistically significant differences were observed across categories of the prudent dietary pattern.

### 3.3. Dietary Patterns and HrHPV Infection

In the age-adjusted model of PCA-derived dietary patterns and hrHPV infection risk, increasing factor scores for the Western pattern were associated with a significantly higher risk of hrHPV infection (OR = 1.44, 95%CI = 1.03–2.03, *p* = 0.036). Particularly, the risk of hrHPV infection was higher among women in Q3 and Q4, than those in Q1 (OR = 1.77, 95%CI = 1.04–3.54, *p* = 0.032 and OR = 1.97, 95%CI = 1.14–4.18, *p* = 0.016, respectively; *p* trend = 0.039) (Table 4). No significant association between the prudent dietary pattern and hrHPV infection was evident. 

Conversely, using *a priori* MDS, we showed that a higher MDS was associated with a significantly lower risk of hrHPV infection in both the age-adjusted (OR = 0.76, 95%CI = 0.64–0.92, *p* = 0.004) and multivariate-adjusted models (OR = 0.79, 95%CI = 0.66–0.96, *p* = 0.018). Although no associations between hrHPV infection and components of the MDS were evident, women with medium adherence to the MD showed a lower risk of hrHPV infection (OR = 0.40, 95%CI = 0.22–0.73, *p* = 0.008; *p* trend = 0.004), compared to low adherents. This result was confirmed after adjustment for age, smoking, nutritional status and parity (OR = 0.40, 95%CI = 0.21–0.75, *p* = 0.010; *p* trend = 0.015) (Table 5). No significant association between high adherence to MD and the risk of hrHPV infection was evident, probably due to the paucity of highly-adherent women.

### 3.4. Dietary Patterns and Cervical Cancer

Table 6 shows the results of the logistic regression analysis of the association between PCA-derived dietary patterns and the risk of CIN2+. With regard to the prudent dietary pattern, the risk was lower among women in Q3 than those in Q1 (OR = 0.50, 95%CI = 0.26–0.98, *p* = 0.039) after adjustment for hrHPV status and age. No statistically significant differences were observed between categories of the Western dietary pattern and adherence to MD (Table 7).

## 4. Discussion

A growing body of evidence suggests that dietary patterns might modulate the risk of female cancers [25,26], and several dietary intervention programs have been proposed to reduce cancer incidence and to improve health and quality of life [27,28,29]. However, to our knowledge, no previous study has evaluated the association between dietary patterns, hrHPV status and high grade CIN risk. Given this lack of evidence, using an *a posteriori* approach, we firstly derived two dietary patterns which characterized the main dietary habits of our study population. The first pattern, named “Western”, was characterized by high intakes of red and processed meats, dipping sauces and chips and snacks, with low intake of olive oil; the second one, named “prudent”, consisted of high intakes of legumes, vegetable soups, potatoes, cooked and raw vegetables. Since the dietary habits of Mediterranean populations have been associated with healthy effects in terms of overall morbidity and mortality [30], we also evaluated the adherence to the MD, using an *a priori* score (i.e., MDS). Interestingly, food groups which positively characterized the prudent dietary pattern in our population reflected some components of MDS (e.g., legumes and vegetables). According to the MDS classification, only 6.9% of enrolled women reported high adherence to MD, which includes a balanced ratio of omega 6 and omega 3 essential fatty acids and high amounts of fiber, antioxidants and polyphenols found in fruit, vegetables, olive oil and wine [31].

Among healthy women, greater adherence to a Western dietary pattern was associated with a higher risk of hrHPV infection. Similarly, a previous study showed that an unhealthy dietary pattern might put women at a higher risk of developing hrHPV-related cervical lesions [32]. This is also in line with results regarding the protective effect of MD, which indicate that a greater MDS is associated with a reduced risk of hrHPV infection. Particularly, compared to low adherents, women with medium adherence to the MD were less likely to be at risk of hrHPV infection. Although the paucity of women with high adherence to MD raises the need of large epidemiological studies investigating this association, recent evidence suggests that several micronutrients typical of MD may help in CC prevention by inhibiting HPV persistence [33]. 

Previous studies have reported that women with lower intakes of vegetables and fruits as well as vitamins A, C, and E have a higher risk of high grade CIN and CC [9,12]. Accordingly, our study highlighted the protective role of the prudent dietary pattern, a Mediterranean-like diet pattern, which was negatively associated with the risk of CIN2+. Particularly, CIN2+ risk was lower among women with medium–high adherence, compared to those with low adherence to the prudent pattern. The biological basis of this evidence could be explained by the activity of dietary factors that may modify the epigenome [34]. The role of epigenetics in women’s health and reproductive functions has been deeply investigated [18,35,36], suggesting bioactive compounds can affect epigenetic signatures by different mechanisms [37]. A study on a cancer free population showed that low fruit consumption and folate deficiency could modulate the Long interspersed nuclear elements 1 (LINE-1) methylation level [36], an epigenetic marker of cancer risk [38]. Particularly, dietary patterns associated with LINE-1 methylation levels could alter the risk of developing cervical intraepithelial neoplasia [32]. Moreover, the consumption of foods which maintain normal DNA methylation levels, such as plant-based foods, could potentially suppress the expression of HPV oncogenes, thereby reducing cell transformation rates and CC risk [3,17]. 

The weaknesses of our study include the cross-sectional design which does not allow determination of causality. Since the use of dietary pattern analysis was largely limited to cross-sectional studies, further large prospective studies should be encouraged. Moreover, food intakes were estimated using the FFQ, which does not preclude measurement error and may suffer from inaccuracies. Nevertheless, the FFQ used in this study was specifically developed for use in our population and was previously validated against a four-day weighted dietary record, with a correlation coefficient in accordance with other FFQ validation studies [20]. Finally, although we adjusted for several factors, known to potentially affect hrHPV status and/or CC risk, we could not rule out the possibility of bias from residual confounding of unmeasured lifestyle and socioeconomic factors. Among these, the relationship between HPV infection and CC risk with sexual behaviors (i.e., early first sexual intercourse, multiple sexual partners, long-term oral contraceptive use) has been widely established. Thus, further studies, investigating the association between dietary patterns and the risk of hrHPV infection and CC, should take into account factors affecting sexual activity and behavior [39]. 

In conclusion, to our knowledge, this study is the first to report evidence regarding the association between dietary patterns and hrHPV infection and CC risk. In the context of CC prevention, our data discourage unhealthy dietary habits in favour of healthy ones such as a Mediterranean-like diet, which reduces the risk of hrHPV infection and CIN2+. However, future prospective large-scale studies are needed to evaluate the association between dietary pattern and CC risk, taking into account the physiological and molecular pathways involved in this relationship.

## Figures and Tables

**Table 1:** Characteristics of healthy women according to high-risk human papillomavirus (hrHPV) status.

Characteristics	hrHPV+ (*n* = 84)	hrHPV– (*n* = 167)	*p*-Value ^a^
Age, mean (SD)	38.63 (10.53)	43.65 (9.62)	**<0.001**
Current smokers	47.0%	28.1%	**0.003**
BMI, mean (SD)	22.36 (4.01)	24.34 (4.68)	**0.001**
Nutritional status			
Underweight	8.4%	5.4%	**0.004**
Normal weight	75.9%	56.6%	
Overweight	10.8%	24.1%	
Obese	4.8%	13.9%	
Workers	51.2%	41.3%	0.138
Parity (≥1 live births)	58.3%	83.2%	**<0.001**
Low education level	31.0%	41.9%	0.092
Use of oral contraceptive	13.1%	7.8%	0.177

SD, standard deviation; BMI, Body Mass Index. ^a^ Statistically significant *p*-values (*p* < 0.05) are indicated in bold font.

**Table 2:** Characteristics of women according to case/control classification.

Characteristics	Cases (*n* = 127)	Controls (*n* = 411)	*p*-Value ^a^
Age, mean (SD)	36.01 (8.10)	41.50 (10.21)	**<0.001**
Current smokers	53.5%	36.8%	**0.001**
BMI, mean (SD)	22.47 (3.63)	23.59 (4.53)	**0.012**
Nutritional status			
Underweight	11.0%	7.1%	0.052
Normal weight	67.5%	63.1%	
Overweight	18.3%	19.8%	
Obese	3.2%	10.0%	
Workers	46.5%	46.5%	0.998
Parity (≥1 live births)	59.8%	71.8%	**0.011**
Low education level	41.0%	38.2%	0.579
Use of oral contraceptive	14.2%	8.0%	0.039

SD, standard deviation; BMI, Body Mass Index. ^a^ Statistically significant *p*-values (*p* < 0.05) are indicated in bold font.

**Table 3:** Characteristics of women according to dietary pattern quartile.

Characteristics	Western Dietary Pattern					Prudent Dietary Pattern				
	Q1 (*n* = 134)	Q2 (*n* = 135)	Q3 (*n* = 135)	Q4 (*n* = 134)	*p*-value ^a^	Q1 (*n* = 134)	Q2 (*n* = 135)	Q3 (*n* = 135)	Q4 (*n* = 134)	*p*-Value ^a^
Age, mean (SD)	42.37 (10.87)	43.35 (9.48)	39.79 (9.25)	35.28 (8.46)	**<0.001**	40.55 (10.74)	40.68 (9.90)	40.52 (9.86)	39.05 (9.58)	0.500
Current smokers	38.1%	34.1%	37.3%	53.7%	**0.005**	41.0%	46.3%	34.8%	41.0%	0.300
BMI, mean (SD)	23.39 (4.23)	23.69 (4.19)	23.76 (4.77)	22.45 (4.13)	0.053	23.18 (4.38)	23.14 (4.30)	23.44 (4.30)	23.53 (4.48)	0.846
Workers	36.6%	50.4%	47.4%	54.1%	0.631	44.8%	45.2%	51.1%	44.8%	0.667
Parity (≥1 live births)	71.5%	77.8%	66.7%	59.7%	**0.011**	63.4%	68.9%	70.4%	73.1%	0.372
Low Education level	43.3%	34.8%	34.1%	43.3%	0.218	40.3%	35.6%	41.5%	38.1%	0.762
Use of oral contraceptive	9.0%	8.9%	11.1%	9.0%	0.906	11.9%	7.4%	9.6%	9.0%	0.644

Q, quartile; SD, standard deviation; BMI, Body Mass Index. ^a^ Statistically significant *p*-values (*p* < 0.05) are indicated in bold font.

**Table 4:** Associations between dietary patterns derived by principal component analysis and the risk of hrHPV infection.

Dietary Pattern	Regression Model	adjOR (95%CI)						
		Q1	Q2	Q3	Q4	*p-*Trend	Continuous	*p*-Value
Western	Model 1 ^a^	1.00 (ref)	1.11 (0.48–2.57)	**1.77 (1.04–3.54)**	**1.97 (1.14–4.18)**	**0.039**	**1.44 (1.03–2.03)**	**0.036**
	Model 2 ^b^	1.00 (ref)	1.33 (0.54–3.28)	1.96 (0.88–4.34)	2.06 (0.86–4.90)	**0.047**	1.39 (0.97–1.99)	0.069
Prudent	Model 1 ^a^	1.00 (ref)	1.18 (0.54–2.60)	0.86 (0.58–1.28)	0.85 (0.65–1.11)	0.842	0.83 (0.63–1.11)	0.215
	Model 2 ^b^	1.00 (ref)	1.05 (0.45–2.43)	0.86 (0.57–1.32)	0.82 (0.62–1.10)	0.226	0.83 (0.62–1.11)	0.210

adjOR, adjusted Odds Ratio; CI, Confidence Interval; Q, quartile. Statistically significant results (*p* < 0.05) are indicated in bold font. ^a^ Adjusted for age; ^b^ Adjusted for age, BMI, smoking status and parity.

**Table 5:** Association between adherence to the Mediterranean diet and the risk of hrHPV infection.

Regression Model	adjOR (95%CI)					
	Low Adherence	Medium Adherence	High Adherence	*p*-Trend	MDS (Continuous)	*p*-Value
Model 1 ^a^	1.00 (ref)	**0.40 (0.22–0.73)**	0.43 (0.15–1.22)	**0.006**	**0.76 (0.64–0.92)**	**0.004**
Model 2 ^b^	1.00 (ref)	**0.40 (0.21–0.75)**	0.50 (0.17–1.50)	**0.015**	**0.79 (0.66–0.96)**	**0.018**

adjOR, adjusted Odds Ratio; CI, Confidence Interval; MDS, Mediterranean Diet Score. Statistically significant results (*p* < 0.05) are indicated in bold font. ^a^ Adjusted for age; ^b^ Adjusted for age, BMI, smoking status and parity.

**Table 6:** Association between dietary patterns derived by principal component analysis and the risk of CIN2+.

Dietary Pattern	Regression Model	adjOR (95%CI)						
		Q1	Q2	Q3	Q4	*p*-Trend	Continuous	*p*-Value
Western	Model 1 ^a^	1.00 (ref)	1.35 (0.73–2.51)	1.05 (0.57–1.94)	1.35 (0.74–2.45)	0.560	1.11 (0.92–1.35)	0.281
	Model 2 ^b^	1.00 (ref)	1.28 (0.63–2.60)	0.90 (0.45–1.80)	1.04 (0.53–2.03)	0.753	1.00 (0.81–1.23)	0.990
Prudent	Model 1 ^a^	1.00 (ref)	0.66 (0.36–1.22)	0.57 (0.31–1.04)	0.77 (0.42–1.40)	0.352	0.87 (0.71–1.05)	0.144
	Model 2 ^b^	1.00 (ref)	0.62 (0.32–1.22)	**0.50** **(0.26–0.98)**	0.58 (0.29–1.14)	0.076	0.83 (0.62–1.11)	0.210

adjOR, adjusted Odds Ratio; CI, Confidence Interval; Q, quartile. Statistically significant results (*p* < 0.05) are indicated in bold font. ^a^ Adjusted for age; ^b^ Adjusted for age and hrHPV status.

**Table 7:** Association between adherence to Mediterranean diet and the risk of CIN2+.

Regression Model	adjOR (95%CI)					
	Low Adherence	Medium Adherence	High Adherence	*p*-Trend	MDS	*p*-Value
Model 1 ^a^	1.00 (ref)	1.01 (0.65–1.57)	0.50 (0.18–1..40)	0.473	1.01 (0.88–1.15)	0.941
Model 2 ^b^	1.00 (ref)	1.23 (0.76–2.01)	0.76 (0.24–2.41)	0.726	1.09 (0.94–1.27)	0.272

adjOR, adjusted Odds Ratio; CI, Confidence Interval. ^a^ Adjusted for age; ^b^ Adjusted for age and hrHPV status.
